# Predictors for early cardiac death after discharge from successfully treated acute myocardial infarction

**DOI:** 10.3389/fmed.2023.1165400

**Published:** 2023-06-15

**Authors:** Young Choi, Kwan Yong Lee, Sang Hyun Kim, Kyung An Kim, Byung-Hee Hwang, Eun Ho Choo, Sungmin Lim, Chan Jun Kim, Jin-Jin Kim, Jaeho Byeon, Gyu Chul Oh, Doo Soo Jeon, Ki Dong Yoo, Ha-Wook Park, Min Chul Kim, Youngkeun Ahn, Myung Ho Jeong, Youngdeok Hwang, Kiyuk Chang

**Affiliations:** ^1^Cardiovascular Center and Cardiology Division, Seoul St. Mary's Hospital, The Catholic University of Korea, Seoul, Republic of Korea; ^2^Cardiovascular Research Institute for Intractable Disease, College of Medicine, The Catholic University of Korea, Seoul, Republic of Korea; ^3^Cardiovascular Center and Cardiology Division, Uijeongbu St. Mary's Hospital, The Catholic University of Korea, Uijeongbu, Republic of Korea; ^4^Cardiovascular Center and Cardiology Division, Incheon St. Mary's Hospital, The Catholic University of Korea, Incheon, Republic of Korea; ^5^Cardiovascular Center and Cardiology Division, St. Vincent's Hospital, The Catholic University of Korea, Suwon, Republic of Korea; ^6^Department of Cardiology, Bucheon Sejong Hospital, Bucheon, Republic of Korea; ^7^Department of Cardiology, Cardiovascular Center, Chonnam National University Hospital, Gwangju, Republic of Korea; ^8^Paul H. Chook Department of Information Systems and Statistics, Baruch College, City University of New York, New York, NY, United States

**Keywords:** death, sudden, cardiac, prognostic factors, myocardial infarction, heart failure, systolic, cardiogenic shock

## Abstract

**Background:**

The use of a cardioverter defibrillator for the primary prevention of sudden cardiac death is not recommended within 40 days after acute myocardial infarction (AMI). We investigated the predictors for early cardiac death among patients who were admitted for AMI and successfully discharged.

**Methods:**

Consecutive patients with AMI were enrolled in a multicenter prospective registry. Among 10,719 patients with AMI, 554 patients with in-hospital death and 62 patients with early non-cardiac death were excluded. Early cardiac death was defined as a cardiac death within 90 days after index AMI.

**Results:**

Early cardiac death after discharge occurred in 168/10,103 (1.7%) patients. A defibrillator was not implanted in all patients with early cardiac death. Killip class ≥3, chronic kidney disease stage ≥4, severe anemia, cardiopulmonary support usage, no dual antiplatelet therapy at discharge, and left ventricular ejection fraction (LVEF) ≤35% were independent predictors for early cardiac death. The incidence of early cardiac death according to the number of factors added to LVEF criteria in each patient was 3.03% for 0 factor, 8.11% for 1 factor, and 9.16% for ≥2 factors. Each model that sequentially added the factors in the presence of LVEF criteria showed a significant gradual increase in predictive accuracy and an improvement in reclassification capability. A model with all factors showed C-index 0.742 [95% CI 0.702–0.781], *p* < 0.001; IDI 0.024 [95% CI 0.015–0.033], *p* < 0.001; and NRI 0.644 [95% CI 0.492–0.795], *p* < 0.001.

**Conclusion:**

We identified six predictors for early cardiac death after discharge from AMI. These predictors would help to discriminate high-risk patients over current LVEF criteria and to provide an individualized therapeutic approach in the subacute stage of AMI.

## Introduction

Acute myocardial infarction (AMI) can result in an ischemic ventricular scar formation, which entails the risk of subsequent ventricular arrhythmias and sudden cardiac death (SCD) (1, 2). Previous studies have reported that the major risk factor for SCD after AMI reduced left ventricular ejection fraction (LVEF) (1, 3) and have shown that prophylactic use of an implantable cardioverter defibrillator (ICD) can provide primary prevention of SCD in high-risk patients (4, 5). However, ICD implantation early after AMI did not result in reduced mortality in two randomized trials (6, 7). Based on these studies, current guidelines recommend the use of prophylactic ICDs in patients who meet the criteria, including LVEF and heart failure symptoms after 6–12 weeks from the AMI event (8).

However, a substantial risk of SCD also exists during the subacute stage of AMI. Previous studies have shown that an incidence of SCD ranges between 0.5 and 3% within 90 days after AMI (1, 6, 9). However, the use of ICD based solely on LVEF during MI hospitalization did not improve survival, which was owing to a non-significant reduction in SCD and increased non-arrhythmic death in the ICD group (6, 7). The causes of SCD during the subacute stage after MI include residual myocardial infarction, altered sympathetic activity, myocardial vulnerability due to stunning, electrolyte imbalances, myocardial inflammation, and stent thrombosis (10–12). Risk prediction would be improved when appropriate variables that reflect the causal mechanisms of SCD at a subacute stage after AMI are integrated with LVEF criteria. Early SCD occurs before a clinician can identify primary prevention indications for ICD, and the efforts to discriminate high-risk patient groups would guide the application of intensive monitoring and preventive strategy for a selective population in this period. Although indiscriminate defibrillator implantation was not beneficial, this individualized approach may further improve early clinical outcomes after AMI (13). In this study, we aimed to identify the predictors that can discriminate patients who are at risk of cardiovascular death within 3 months after discharge from an AMI event, using large-scale multicenter data.

## Materials and methods

### Study protocols and population selection

The COREA-AMI registry, designed to evaluate the long-term clinical outcomes of AMI patients, examined subjects from a total of nine major cardiac centers located in urban areas throughout Korea. Each center regularly performs a high volume of percutaneous coronary intervention (PCI) procedures. Split into two parts, the COREA-AMI I registry included AMI patients who underwent PCI between January 2004 and December 2009, while the COREA-AMI II registry included an extended follow-up of COREA-AMI I patients as well as newly enrolled AMI patients between January 2010 and August 2014. All clinical, angiographic, and follow-up data of these AMI patients were sequentially registered in a web-based case reporting system. The COREA-AMI study was approved by the Institutional Review Board (IRB), conducted in adherence to the Declaration of Helsinki, and executed according to the guidelines of STROBE (14). The registry is registered on ClinicalTrials.gov (study ID: NCT02806102).

**Figure 1:**
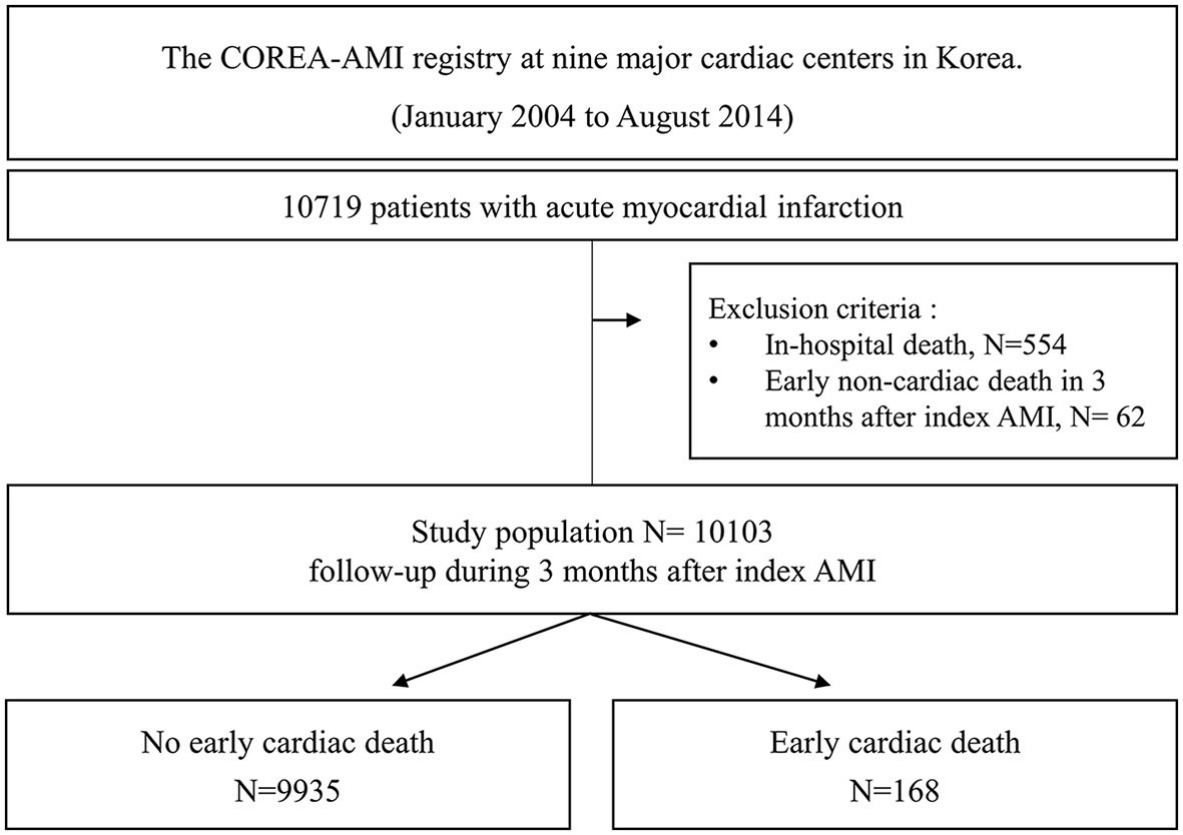
Study flowchart.

A total of 10,719 AMI patients who received drug-eluting stent implantations were enrolled in the registry, and 554 patients with in-hospital death and 62 patients with early non-cardiac death were excluded. Finally, a total of 10,103 patients were included in the analysis of this study. A study flowchart is depicted in Figure 1. The patients were categorized into two groups according to the occurrence of early cardiac death within 3 months.

### Treatment and data collection

Among 10,103 patients, 9,769 patients received PCI treatment within 48 h of admission, with coronary artery angiography (CAG) and primary PCI both performed in adherence to standard guidelines. Among the remaining 334 patients, 14 patients received only thrombus aspiration, 30 patients received drug-eluting balloon, 274 patients received only balloon angioplasty, and 16 patients were medically treated. The coronary disease was considered significant if the epicardial coronary arteries had angiographic stenosis ≥70% and if the left main coronary artery had stenosis ≥50%. Loading doses of the antiplatelet agents (aspirin, 300 mg; clopidogrel, 300 or 600 mg; cilostazol, 200 mg; ticagrelor, 180 mg; or prasugrel, 60 mg) were prescribed for all patients before or during PCI. Patients with DES were prescribed 100 mg of aspirin daily and/or a P2Y12 inhibitor (75 mg of clopidogrel once daily, 90 mg of ticagrelor twice daily, or 10 mg of prasugrel once daily). The duration of dual antiplatelet therapy (DAPT) was determined by a physician in accordance with the final diagnosis at baseline and the complexity of the revascularization procedure. The post-intervention medications included aspirin, clopidogrel, statins, angiotensin-converting enzyme inhibitors (ACEi) or angiotensin II receptor blockers (ARBs), and β-blockers. These medications were administered within 24 h of PCI and, unless contraindicated, were continued after discharge. Each physician used their own judgment when choosing to perform predilatation, direct stenting, post-adjunct balloon inflation, or administering glycoprotein IIb/IIIa receptor.

Trained reviewers who were blinded to results then gathered relevant patient data using hospital chart reviews and phone interviews and, after removing personally identifiable information, organized the data into a web-based system. These data included follow-up, survival, and clinical event data and were collected through 31 March 2019. Electronic medical records and phone interviews were similarly used to evaluate clinical events and outcome data. Angiographic and procedural data were evaluated by independent reviewers and interventional cardiologists, while independent research personnel gathered baseline, clinical, laboratory, and medication data. Any adverse clinical events of interest were confirmed by the committee of the Cardiovascular Center of Seoul St. Mary's Hospital, and mortality was confirmed based on disqualification from the National Health Insurance Service, Korea's single-payer, universal healthcare program. Independent statisticians at the clinical research coordinating center handled the final dataset, with the clinical research associate sealing it with a code.

### Study endpoint and definition

The main study outcome was the occurrence of early cardiac death. Cardiac death was defined as a death due to AMI, heart failure, cardiac arrhythmias, other cardiovascular causes, or an unexpected sudden death without an obvious non-cardiac cause. Early cardiac death was defined as a cardiac death within 90 days after the date of AMI diagnosis.

### Statistical analysis

Categorical variables were presented as numbers and relative frequencies (percentages) and were compared using the chi-squared test or Fisher's exact test. Continuous variables were expressed as the mean ± standard deviation or median (Q1, Q3), depending on whether they were normally distributed or not, and were compared using the independent-samples *t*-test or Mann–Whitney *U*-test, as appropriate. To identify independent predictors of early cardiac death, we used a multivariable Cox proportional hazards model. The adjusted variables for the multivariate model were selected if they were significantly different between the two groups (*p* < 0.05 in the univariate analysis) for the baseline clinical and procedural characteristics. The adjusted variables for the multivariate Cox proportional hazards regression analysis were as follows: age ≥75 years, female, severe left ventricle (LV) dysfunction (ejection fraction, EF ≤35%), KILLIP III or IV, chronic kidney disease (CKD) stage ≥4 [estimated glomerular filtration rate (eGFR) <30 ml/min/1.73 m^2^], severe anemia (hemoglobin, Hb <11 g/dL), extracorporeal membrane oxygenation (ECMO) or intra-aortic balloon pump (IABP), cardiopulmonary resuscitation (CPR) before treatment, atrial fibrillation, hypertension, previous stroke, chronic lung disease, left main PCI, complete revascularization, and no DAPT at discharge. The cumulative early cardiac death event rates of each group stratified according to the number of risk factors were calculated using a Kaplan-Meier estimator and compared using the log-rank statistic. Unadjusted hazard ratios during 90 days from index AMI were determined from Cox proportional hazards models.

In addition, various prediction models were constructed to assess the incremental prognostic value of adding observed predictors to severe LV dysfunction, a conventional risk factor of early cardiac death and the only indication of ICD insertion: (1) Conventional risk factor model: severe LV dysfunction; (2) novel risk factors combination models: severe LV dysfunction + each of or gradually added novel risk factors (ECMO or IABP, KILLIP III or IV, severe anemia, CKD stage ≥4, no DAPT at discharge) except old age. The discriminative ability of the models was assessed using Harrell's C-index, which is analogous to the area under the receiver operator characteristic curve and was applied to all-cause mortality data. The receiver operating characteristic (ROC) curves in logistic regression were used. Reclassification performance was compared using the relative integrated discrimination improvement (IDI) and continuous net reclassification index (NRI). Larger relative IDI values indicate greater improvements in model discrimination (15). Improvements in subject risk reclassification were further assessed using continuous NRI and were applied to the early cardiac death data. Each measure was analyzed using R version 4.1.2 (R Foundation for Statistical Computing, Vienna, Austria). The survival package in R was used for survival analysis. The pROC package was used to interpret a ROC curve in logistic regression in R. Statistical significance was indicated by a two-tailed *p*-value of < 0.05.

## Results

### Baseline patient characteristics

A total of 10,103 post-AMI survivors who were successfully discharged after in-hospital care were analyzed. The baseline clinical, procedural, and laboratory characteristics and medication at discharge information are listed in Tables 1, 2. The mean age of all the included patients was 63.1 ± 12.7 years. Overall, 31.1% of the patients had diabetes, 51.7% had hypertension, 4.1% had a previous MI, 5.1% had CKD stage ≥4, 96.7% had PCI, 53.5% had multivessel PCI, 13.0% showed KILLIP III or IV presentations during admission, and 2.3% of the patients required hemodynamic cardiopulmonary support device use. The mean LVEF was 53.4 ± 11.1%, and 6.4% had severe LV dysfunction. A total of 53.6% presented with ST-segment elevation MI.

**Table 1:** Baseline clinical and procedural characteristics.

	**Total (*n* = 10,103)**	**No early cardiac death (*n* = 9,935)**	**Early cardiac death (*n* = 168)**	***p*-value**
**Clinical characteristics**				
Age, year	63.1 ± 12.7	62.9 ± 12.6	74.8 ± 10.3	< 0.001
≥75	2,097 (20.8)	2,003 (20.2)	94 (56.0)	< 0.001
Female	2,791 (27.6)	2,721 (27.4)	70 (41.7)	< 0.001
DM	3,147 (31.1)	3,079 (31.0)	68 (40.5)	0.893
HBP	5,224 (51.7)	5,111 (51.4)	113 (67.3)	0.011
Dyslipidemia	1,632 (16.2)	1,606 (16.2)	26 (15.5)	0.893
History of Stroke	701 (6.9)	684 (6.9)	17 (10.1)	0.138
Previous MI	412 (4.1)	402 (4.0)	10 (6.0)	0.297
Previous PCI	722 (7.1)	706 (7.1)	16 (9.5)	0.291
Previous CABG	51 (0.5)	48 (0.5)	3 (1.8)	0.053
Cancer	321 (3.2)	316 (3.2)	5 (3.0)	1
Chronic lung disease	237 (2.3)	227 (2.3)	10 (6.0)	0.006
Chronic liver disease	91 (0.9)	89 (0.9)	2 (1.2)	0.665
Atrial fibrillation on baseline ECG	309 (3.1)	298 (3.0)	11 (6.5)	0.015
eGFR < 30 ml/min/1.73 m^2^	518 (5.1)	481 (4.8)	37 (22.0)	< 0.001
Severe anemia (Hb < 11 g/dL)	1,886 (18.7)	939 (9.5)	50 (29.8)	< 0.001
LVEF	53.4 ± 11.1	53.6 ± 11.0	44.3 ± 12.6	< 0.001
LVEF ≤ 35%	650 (6.4)	611 (6.1)	39 (23.2)	< 0.001
KILLIP III or IV	1,316 (13.0)	1,256 (12.6)	60 (35.7)	< 0.001
CPR before treatment	229 (2.3)	220 (2.2)	9 (5.4)	0.014
ST-segment elevation MI	5,419 (53.6)	5,339 (53.7)	80 (47.6)	0.134
**Procedural characteristics**				
PCI	9,769 (96.7)	9,608 (96.7)	161 (95.8)	0.681
Total stent number	1.6 ± 0.9	1.6 ± 0.9	1.6 ± 0.9	0.799
Left main as target lesion	358 (3.5)	345 (3.5)	13 (7.7)	0.006
Bifurcation with two stents (non-LM)	154 (1.5)	151 (1.5)	3 (1.8)	0.744
Restenosis lesion	164 (1.6)	161 (1.6)	3 (1.8)	0.755
Multivessel PCI	5,403 (53.5)	5,301 (53.4)	102 (60.7)	0.069
CTO lesion	509 (5.0)	502 (5.1)	7 (4.2)	0.732
Long stenting (>60 mm)	435 (4.3)	427 (4.3)	8 (4.8)	0.919
Complete revascularization	6,719 (66.5)	6,623 (66.7)	96 (57.1)	0.012
At least three stents implanted	1,472 (14.6)	1,447 (14.6)	25 (14.9)	0.996
At least three lesions treated	455 (4.5)	445 (4.5)	10 (6.0)	0.468
Second-generation DES	6,426 (63.6)	6,320 (63.6)	106 (63.1)	0.954
ECMO/IABP	231 (2.3)	219 (2.2)	12 (7.1)	< 0.001

Data are presented as n (%) for categorical variables and mean ± standard deviation for continuous variables. eGFR = 141 × min (Scr/κ,1)α × max (Scr/κ, 1) – 1.209 × 0.993 Age × 1.018 (if female) × 1.159 (if black). Scr is serum creatinine (mg/dL), κ is 0.7 for women and 0.9 for men, α is – 0.329 for women and – 0.411 for men, min indicates the minimum of Scr/κ or 1, and max indicates the maximum of Scr/κ or 1.

DM, diabetes mellitus; HBP, high blood pressure; MI, myocardial infarction; PCI, percutaneous coronary intervention; CABG, coronary artery bypass graft; ECG, electrocardiography; eGFR, estimated glomerular filtration rate; Hb, hemoglobin; LVEF, left ventricle ejection fraction; LM, left main; CTO, chronic total occlusion; DES, drug-eluting stent; ECMO, extracorporeal membrane oxygenation; IABP, intra-aortic balloon pump.

**Table 2:** Baseline laboratory findings and medication characteristics.

	**Total (*n* = 10,103)**	**No early cardiac death (*n* = 9,935)**	**Early cardiac death (*n* = 168)**	***p*-value**
**Laboratory findings**				
CK-MB, peak, ng/mL	60.4 (16.2, 171.3)	60.4 (16.2, 171.9)	38.3 (15.4, 129.0)	< 0.001
Total cholesterol, mg/dL	178.2 ± 43.4	178.5 ± 43.3	158.8 ± 44.4	< 0.001
Triglyceride, mg/dL	124.9 ± 92.7	125.2 ± 93.1	102.9 ± 60.8	< 0.001
High-density lipoprotein, mg/dL	40.9 ± 10.9	41.0 ± 10.9	39.8 ± 11.6	0.226
Low-density lipoprotein, mg/dL	113.7 ± 37.9	113.9 ± 37.8	97.1 ± 39.2	< 0.001
High-sensitivity CRP, mg/dL	6.8 ± 35.4	6.7 ± 35.4	16.9 ± 35.4	0.001
**Medication at discharge**				
Dual antiplatelet therapy	9,888 (97.9)	9,736 (98.0)	152 (90.5)	< 0.001
Aspirin	9,935 (98.3)	9,780 (98.4)	155 (92.3)	< 0.001
Clopidogrel	8,702 (86.1)	8,558 (86.1)	144 (85.7)	0.964
Potent P2Y12 inhibitor	1,343 (13.3)	1,331 (13.4)	12 (7.1)	0.024
Beta-blocker	8,852 (87.6)	8,709 (87.7)	143 (85.1)	0.382
ACEi or ARB	7,886 (78.1)	7,772 (78.2)	114 (67.9)	0.002
Oral anticoagulant	250 (2.5)	243 (2.4)	7 (4.2)	0.2
Statin	9,734 (96.3)	9,575 (96.4)	159 (94.6)	0.327

Data are presented as n (%) for categorical variables and mean ± standard deviation for continuous variables. Peak CK-MB level is presented as median (Q1, Q3). Dual antiplatelet therapy combines aspirin and a P2Y12 inhibitor (clopidogrel or potent P2Y12 inhibitor).

CK-MB, creatinine kinase MB isoenzyme; CRP, C-reactive protein; ACEi, angiotensin-converting enzyme inhibitors; ARB, angiotensin II receptor blockers.

Of the 10,103 patients, early cardiac death after discharge occurred in 168 (1.7%) patients (Table 1). The patients with early cardiac death were more likely to be older, female, with hypertension, chronic lung disease, atrial fibrillation, CKD stage ≥4, severe anemia, impaired LV systolic function, KILLIP III or IV, and had CPR before treatment. There were more left main PCI and use of cardiopulmonary supports. Regarding laboratory data, the patients with early cardiac death had higher levels of total cholesterol, triglyceride, low-density lipoprotein, and high-sensitivity C-reactive protein than those without early cardiac death (Table 2). The former used aspirin or potent P2Y12 inhibitor, ACEi or ARB less often as a discharge medication. An ICD was implanted within 90 days after AMI in six (0.06%) patients without early cardiac death, and in no patients with early cardiac death.

### Independent predictors on the risk of early cardiac death in AMI

**Figure 2:**
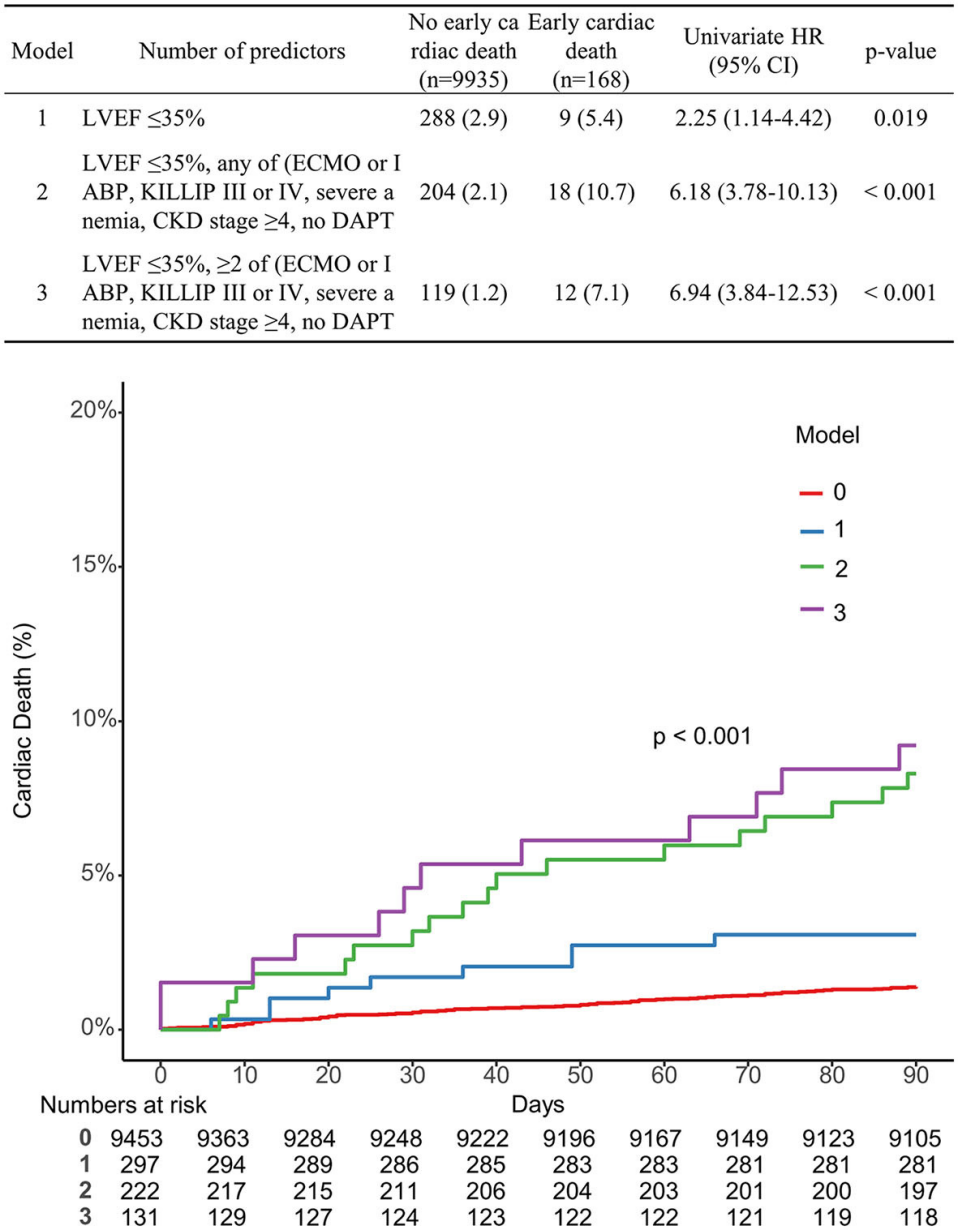
Comparison of the K-M curve according to the number of predictors associated with early cardiac death.

Multivariable Cox proportional hazard models identified independent predictors of early cardiac death (Table 3). Severe LV dysfunction, old age ≥ 75, KILLIP class III or IV, CKD stage ≥4, severe anemia, ECMO or IABP usage, and no DAPT at discharge were independently associated with a decreased risk of early cardiac death (adjusted hazard ratio (HR): 2.55, 95% CI: 1.75–3.71, *p* < 0.001; adjusted HR: 3.58, 95% CI: 2.58–4.98, *p* < 0.001; adjusted HR: 1.78, 95% CI: 1.24–2.55, *p* = 0.002; adjusted HR: 2.41, 95% CI: 1.59–3.66, *p* < 0.001; adjusted HR: 1.65, 95% CI: 1.13–2.41, *p* = 0.01; adjusted HR: 2.0, 95% CI: 1.10–3.76, *p* = 0.023; adjusted HR: 4.16, 95% CI: 2.47–6.99, *p* < 0.001, respectively; Table 3). The K-M estimated early cardiac death rate was significantly higher when the population had more numbers of risk factors (*p* < 0.001; Figure 2). The incidence of early cardiac death according to the number of the novel risk factors (ECMO or IABP, KILLIP III or IV, severe anemia, CKD stage ≥4, no DAPT at discharge) added to a conventional risk factor (severe LV dysfunction) in each patient was 3.03% for 0 factor, 8.11% for 1 factor, and 9.16% for ≥2 factors. Univariate HR (95% CI) is depicted in Figure 2; Model 1 with severe LV dysfunction: 2.25 (1.14–4.42), *p* = 0.019; Model 2 with severe LV dysfunction + any of novel risk factors: 6.18 (3.78–10.13), *p* < 0.001; and Model 3 with severe LV dysfunction + ≥2 of novel risk factors: 6.94 (3.84–12.53), *p* < 0.001.

**Table 3:** Impact of clinical and procedural predictors on the risk of early cardiac death in acute myocardial infarction patients.

	**No early cardiac death (*n* = 9,935)**	**Early cardiac death (*n* = 168)**	**Unadjusted**		**Multivariable-adjusted**	
			**HR (95% CI)**	***p*** **-value**	**HR (95% CI)**	***p*** **-value**
Severe LV dysfunction (EF ≤ 35%)	611 (6.1)	39 (23.2)	4.51 (3.15–6.46)	< 0.001	2.55 (1.75–3.71)	< 0.001
KILLIP III or IV	1,256 (12.6)	60 (35.7)	3.77 (2.75–5.16)	< 0.001	1.78 (1.24–2.55)	0.002
CKD stage ≥ 4 (eGFR < 30 ml/min/1.73 m^2^)	481 (4.8)	37 (22.0)	5.43 (3.77–7.83)	< 0.001	2.41 (1.59–3.66)	< 0.001
Severe anemia (Hb < 11 g/dL)	939 (9.5)	50 (29.8)	3.97 (2.85–5.52)	< 0.001	1.65 (1.13–2.41)	0.01
ECMO or IABP	219 (2.2)	12 (7.1)	3.37 (1.88–6.07)	< 0.001	2.04 (1.10–3.76)	0.023
No dual antiplatelet therapy at discharge	199 (2.0)	16 (9.5)	5.16 (3.08–8.63)	< 0.001	4.16 (2.47–6.99)	< 0.001
Old age ≥ 75	2,003 (20.2)	94 (56.0)	4.9 (3.61–6.65)	< 0.001	3.58 (2.58–4.98)	< 0.001

Values are number of events (%) unless otherwise indicated. The variables of multivariate analysis: severe LV dysfunction (EF ≤ 35%), old age ≥ 75, KILLIP III or IV, eGFR ≤ 30, severe anemia (< 11 g/dL), ECMO or IABP, female, CPR before treatment, atrial fibrillation, hypertension, previous stroke, chronic lung disease, left main PCI, complete revascularization, and no dual antiplatelet therapy at discharge.

HR, hazard ratio; CI, confidence interval; LV, left ventricle; EF, ejection fraction; CKD, chronic kidney disease; Hb, hemoglobin; other abbreviations as in Table 1.

### Prognostic power of risk factors combination predicting early cardiac death

**Figure 3:**
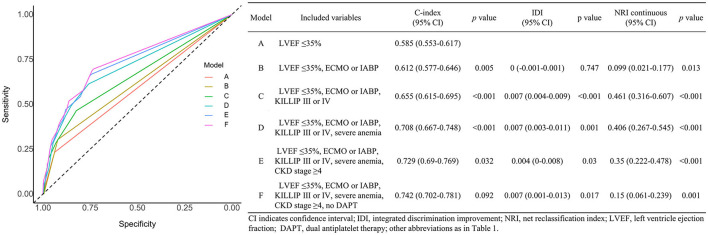
Effects of variables associated with early cardiac death on the prediction accuracy and risk reclassification of each model.

A receiver operating characteristic analysis was performed to evaluate the availability of the risk factors to predict early cardiac death in patients with AMI. A model with all risk factors except age ≥75 showed a significant increase in prognostic performance over a conventional risk factor (severe LV dysfunction): C-index 0.742 [95% CI 0.702–0.781], *p* < 0.001; IDI 0.024 [95% CI 0.015–0.033], *p* < 0.001; and NRI 0.644 [95% CI 0.492–0.795], *p* < 0.001. Figure 3 shows the incremental value of the novel risk factors (ECMO or IABP, KILLIP III or IV, severe anemia, CKD stage ≥4, no DAPT at discharge) over a conventional risk factor using model global performance. Each model that sequentially adds the novel risk factors in the presence of the conventional risk factor showed a significant gradual increase in predictive accuracy and an improvement in reclassification capability. Model A with severe LV dysfunction showed a C-index of 0.585 [95% CI 0.553–0.617]. Model B with severe LV dysfunction + ECMO or IABP showed a C-index of 0.612 [95% CI 0.577–0.646], *p* = 0.005, and NRI 0.099 [95% CI 0.021–0.177], *p* = 0.013. Model C with severe LV dysfunction + ECMO or IABP and KILLIP III or IV showed a C-index of 0.655 [95% CI 0.615–0.695], *p* < 0.001; IDI 0.007 [95% CI 0.004–0.009], *p* < 0.001; and NRI 0.461 [95% CI 0.316–0.607], *p* < 0.001. Model D with severe LV dysfunction + ECMO or IABP, KILLIP III or IV, and severe anemia showed a C-index of 0.708 [95% CI 0.667–0.748], *p* < 0.001; IDI 0.007 [95% CI 0.003–0.011], *p* = 0.001; and NRI 0.406 [95% CI 0.267–0.545], *p* < 0.001. Model E with severe LV dysfunction + ECMO or IABP, KILLIP III or IV, severe anemia, and CKD stage ≥4 showed a C-index of 0.729 [95% CI 0.69–0.769], *p* = 0.032; IDI 0.004 [95% CI 0–0.008], *p* = 0.030; and NRI 0.35 [95% CI 0.222–0.478], *p* < 0.001. Model F with severe LV dysfunction + ECMO or IABP, KILLIP III or IV, severe anemia, CKD stage ≥4, and no DAPT at discharge showed a C-index of 0.742 [95% CI 0.702–0.781], *p* = 0.092; IDI 0.007 [95% CI 0.001–0.013], *p* = 0.017; and NRI 0.15 [95% CI 0.061–0.239], *p* = 0.001.

The results were consistent after stratification based on age 75 (Table 4). Models (A2 and B2) with all risk factors showed significantly higher predictive accuracy and an improvement in reclassification capability than conventional risk factor models (A1, B1). In the age < 75 subgroup, Model A2: C-index of 0.744 [95% CI 0.681–0.807], *p* < 0.001; IDI 0.038 [95% CI 0.02–0.057], *p* < 0.001; and NRI 0.853 [95% CI 0.628–1.078], *p* < 0.001. In the age ≥75 subgroup, Model B2: C-index of 0.709 [95% CI 0.654–0.765], *p* < 0.001; IDI 0.015 [95% CI 0.005–0.025], *p* = 0.002; and NRI 0.476 [95% CI 0.27–0.682], *p* < 0.001.

**Table 4:** Prognostic impact of variables associated with early cardiac death on the prediction accuracy and risk reclassification in subgroup population stratified by age.

**Model**	**Included variables**	**C-index (95% CI)**	***p*-value**	**IDI (95% CI)**	***p*-value**	**NRI continuous (95% CI)**	***p-*value**
**Age**<**75**							
A1	LVEF ≤ 35%	0.568 (0.523–0.613)					
A2	LVEF ≤ 35%, ECMO or IABP, KILLIP III or IV, severe anemia, CKD stage ≥ 4, and no DAPT	0.744 (0.681–0.807)	< 0.001	0.038 (0.02–0.057)	< 0.001	0.853 (0.628–1.078)	< 0.001
**Age** ≥**75**							
B1	LVEF ≤ 35%	0.587 (0.541–0.632)					
B2	LVEF ≤ 35%, ECMO or IABP, KILLIP III or IV, severe anemia, CKD stage ≥ 4, and no DAPT	0.709 (0.654–0.765)	< 0.001	0.015 (0.005–0.025)	0.002	0.476 (0.27–0.682)	< 0.001

CI, confidence interval; IDI, integrated discrimination improvement; NRI, net reclassification index; LVEF, left ventricle ejection fraction; DAPT, dual antiplatelet therapy at discharge; other abbreviations as in Table 1.

## Discussion

In this large-scale, multicenter prospective registry of all-comer AMI patients, early (<90 days) cardiac death occurred in 1.7% of patients who were successfully treated and discharged after AMI. KILLIP class ≥3, chronic kidney disease stage ≥4, severe anemia, cardiopulmonary support usage, no DAPT at discharge, and severe LV dysfunction (LVEF ≤35%) were independently associated with early cardiac death. Currently, LVEF is the most important criterion for discriminating against patients at high risk of SCD. The four factors, except for LVEF in our study, had incremental value in predicting patients at risk of cardiac death during the subacute stage after AMI.

In the VALIANT trial, which enrolled 14,703 patients with AMI and heart failure, the risk of SCD was highest within 30 days after AMI and gradually decreased after 90 days (1). The rate of SCD within 90 days in the VALIANT trial was ~ 3%. In a real-world analysis using a nationwide Swedish registry, the rate of out-of-hospital cardiac arrest (OHCA) within 90 days after AMI was 0.3%, and the non-OHCA death rate was 1.8% (16). The cardiac death rate in our study (1.7% in 90 days) appears to be lower than expected; however, with the consideration of recent advancements in stent technology and heart failure medical therapy, the rate of early mortality may also have decreased compared to previous studies. All patients enrolled in this study underwent an initial invasive approach, and revascularization was performed in 96.7% of patients. Additionally, ~98% of patients received dual antiplatelet therapy, 87.6% received beta-blockers, and 96.3% were taking statins; while the proportion of appropriate medical therapy was high, the proportion of patients with severe LV dysfunction (LVEF ≤35%) was low (6.4%) in our study. Therefore, a better prognosis is expected in our study population compared with previous studies that enrolled patients with AMI and HF. Nevertheless, still significant patients experienced early cardiac death. We obtained five clinical predictors besides LVEF and old age, and among these factors, the KILLIP class and mechanical cardiopulmonary support would reflect the extent of infarcted myocardium, while the renal impairment and anemia factors would reflect the overall comorbidity status of the patient. The results of this study are similar to the factors included in the model for predicting OHCA within 90 days after AMI was performed in the Swedish registry, which concluded that male sex, diabetes, eGFR 30 ml/min/1.73 m^2^, KILLIP class ≥2, new-onset AF, and impaired LVEF were independent predictors (16). In contrast, the female sex was associated with a higher risk of early cardiac death, and diabetes and AF did not show a significant association with early cardiac death in our study. The difference between our study and the Swedish registry study lies in the definition of the patient population and outcome. The population enrolled in our study was older (mean 63 vs. 69), and the revascularization rate was higher in our study (96.7 vs. 78%). The definition of cardiac death in our study was not limited to OHCA, so the early cardiac death possibly included deaths caused by sudden ventricular arrhythmia as well as those caused by pump failure. However, the definition of OHCA in the former study also cannot accurately represent arrhythmic death; any non-arrhythmic cardiac arrest that occurred outside a hospital could have been included as OHCA.

The therapeutic approach to prevent early death after AMI has not been clearly established yet. In the previous studies that showed the survival benefit of ICD, enrollment began at least 30 months after the AMI event (4, 5). The studies which evaluated the effect of early ICD strategy within 30 days after AMI could not prove the effect of ICD on all-cause death, but a decrease in the incidence of SCD was seen in the ICD group (6, 7). With a particular interest in high-risk patients, the DAPA trial studied the benefit of early ICD performed within 1–2 months after AMI in patients with ST-segment elevation AMI and LVEF <30% or poor post-PCI reflow (17). The risk of total death and SCD was significantly lower in the ICD group in the DAPA trial. Conventional ICDs need an invasive implantation procedure, which may increase the risk of procedure-related morbidity. A wearable cardioverter defibrillator can provide short-term protection against SCD more safely (13). The VEST study compared the incidence of SCD in 90 days with and without applying a wearable cardioverter defibrillator (WCD) in patients with AMI and immediate LVEF of 35% or less (9). A total of 2,302 patients enrolled in the study, and 2.4% of arrhythmic deaths occurred in the group without the use of WCD, but also SCD occurred in 1.6% of patients in the WCD group and did not meet statistical significance. Considering the low absolute mortality reduction rate, it would not be cost-beneficial to apply the WCD to all patients using only LVEF as the criterion. If a future study is performed with a selection of a more specific high-risk group that provides intensive education and motivation, a different result may be obtained.

In our study, there were 1,813 (17.9%) patients with LVEF ≤ 35%. Among those, 627 had one additional risk factor besides LVEF, and early cardiac death occurred in 6.5%. In the 202 patients with two additional risk factors, early cardiac death occurred in 10%. Based on these results, intensive monitoring and defibrillator application might be considered for selective patients who account for 10–35% of the patients with low LVEF after AMI. For the mechanism of SCD within 90 days, not only the extent of ventricular ischemic scar and arrhythmogenicity but also myocardial vulnerability due to acute stunning and electrolyte imbalance can be involved (18). Although this study mainly found the clinical factors, additional tests that reflect the myocardial injury and vulnerability would make the prediction more accurate. Nestelberger et al. reported that by incorporating high-sensitivity cardiac troponin measurement at presentation and after 1 h into the prediction algorithm, it is possible to more accurately predict the risk of 30-day major adverse cardiac events (19). Rizas et al. (10) assessed periodic repolarization dynamics (PRD) in 20-min ECG recordings of 450 AMI survivors and found that PRD was a strong and independent predictor for mortality after AMI. In the current study, those factors were not routinely assessed, and a single baseline troponin test was not a significant predictor for early cardiac death. However, utilization of other tests that can be performed after AMI, such as signal-averaged Holter, cardiac imaging, and functional examinations, in addition to clinical factors, could increase the predictive accuracy of early mortality (20–22). This effort would be necessary to find an effective way to reduce early mortality after AMI that cannot be prevented by conventional treatments. The five factors other than LVEF criteria found in this study are easy to identify in routine clinical practice for AMI. The risk of early cardiac death is ~6 times higher in those with low LVEF and having any of the other risk factors. In such patients, more careful observation, evaluation, and educations are necessary, and further research is needed on the treatment strategy that can reduce the subacute mortality rate of these patients.

### Limitations

This study aimed to discriminate against patients who survived the acute phase of AMI but had early SCD that could not be predicted. The study used data from a large prospective AMI registry, but the registry did not distinctively collect the outcome of SCD or arrhythmic death, and the definition of cardiac death was a death due to cardiovascular disease or an unexpected death without an obvious non-cardiac cause. Therefore, early cardiac death defined in this study included deaths due to pump failure as well as SCDs. However, the study patients were those who had been stabilized and discharged after AMI, so the proportion of patients who had fatal pump failure within 3 months would not be high. In addition, the impacts of predictors of early cardiac death identified in our results should be limited to patients who have stabilized after myocardial infarction and successfully discharged. As in-hospital dead patients are excluded, it cannot be extended to all post-myocardial infarction patients. Second, it is thought that the proportion of subacute or late stent thrombosis would substantially contribute to early SCD after AMI, and the patients with stent thrombosis may have different risk factors from patients with pure arrhythmic death. However, it is difficult to completely distinguish between these two deaths. In this study, procedure-related variables did not show a significant association with the outcome, and the prescription rate of the dual antiplatelet agent was sufficiently high in patients with early cardiac death. Third, considering the etiology of early cardiac death after PCI, it is likely that ECG performed at discharge after stabilization AMI can provide valuable information, but the ECG data were not available in the present study and could not be added to the analysis. Fourth, the data on the baseline ICD rate before the AMI event were not available. However, we believe that the number of patients with an implanted ICD at the time of AMI would be negligible, because only a few patients had pre-existing MI or HF at baseline, and the implantation rate of primary prevention ICDs in Korea during the study period was very low (23). Fifth, the study was a non-randomized, retrospective study, which decreased the statistical power to detect differences. No DAPT at discharge was an independent predictor of early cardiac death but rare (about 2.0%) in our cohort. The reason of no DAPT was applied at discharge could not be proven due to the limitation of retrospective study; however, that might reflect the presence of other comorbidities such as high bleeding risks. Our findings need validation from prospectively designed research or randomized controlled trials with large populations in the future. Sixth, although we have presented results by multivariate Cox regression analysis that adjust differences in confounding factors described in the baseline table, differences in results may occur due to differences in unmeasured confounding factors. However, to minimize this difference, we have tried to include as many combinations of factors as possible, including procedural findings that can affect cardiac cause mortality.

## Conclusion

In a large-scale, prospective, multicenter registry data of AMI patients, we found five factors (Killip class ≥ 3, chronic kidney disease stage ≥ 4, severe anemia, cardiopulmonary support usage, no DAPT at discharge, and LVEF ≤ 35%) that were independently associated with cardiac death within 90 days. Even in patients who are successfully treated and discharged after AMI, there remains a residual risk of death in the subacute stage. The current indication of ICD for the primary prevention of SCD is unable to cover this risk. Therefore, further research is needed to develop an approach that can more accurately predict the patients at high risk to consider selective intensive outpatient monitoring or transient defibrillator application.

## Data availability statement

The raw data supporting the conclusions of this article will be made available by the authors, without undue reservation.

## Ethics statement

The studies involving human participants were reviewed and approved by the Catholic Medical Center Central Institutional Review Board. Written informed consent for participation was not required for this study in accordance with the national legislation and the institutional requirements.

## Author contributions

YC contributed to the conceptualization, methodology, writing—original draft, preparation and visualization, and writing—review and editing. KL contributed to the formal analysis, writing—original draft, writing—review and editing, supervision, and project administration. SK, KK, and B-HH helped with validation, investigation, and resources. EC helped with the formal analysis. SL and CK helped with the investigation. J-JK, JB, GO, DJ, KY, MK, YA, MH, and KC helped with resources. KC helped with data curation and project administration. All authors critically revised the manuscript and approved the final version of the manuscript.
